# *In Vitro* Activity of *β*-Lactams in Combination with *β*-Lactamase Inhibitors against* Mycobacterium tuberculosis* Clinical Isolates

**DOI:** 10.1155/2018/3579832

**Published:** 2018-07-02

**Authors:** Fu Li, Li Wan, Tongyang Xiao, Haican Liu, Yi Jiang, Xiuqin Zhao, Ruibai Wang, Kanglin Wan

**Affiliations:** ^1^State Key Laboratory for Infectious Diseases Prevention and Control, Collaborative Innovation Center for Diagnosis and Treatment of Infectious Diseases, National Institute for Communicable Disease Control and Prevention, Chinese Center for Disease Control and Prevention, Beijing 102206, China; ^2^Department of Physiology, Xiangya School of Medicine, Central South University, Changsha, Hunan 410078, China

## Abstract

**Objectives:**

Evaluating the activity of nineteen *β*-lactams in combination with different *β*-lactamase inhibitors to determine the most potent combination against* Mycobacterium tuberculosis* (MTB)* in vitro*.

**Methods:**

Drug activity was examined by drug susceptibility test with 122 clinical isolates from China. Mutations of* blaC* and drug targets* ldt*_*Mt1*_,* ldt*_*Mt2*_,* dacB2,* and* crfA *were analyzed by nucleotide sequencing.

**Results:**

Tebipenem (TBM) in combination with clavulanate (CLA) exhibited the highest anti-TB activity. The MIC of *β*-lactam antibiotics was reduced most evidently in the presence of CLA, compared to avibactam (AVI) and sulbactam (SUB). Eight polymorphism sites were identified in* blaC*, which were not associated with *β*-lactams resistance. Interestingly, one strain carrying G514A mutation in* blaC* was highly susceptible to *β*-lactams regardless of the presence of inhibitors. The transpeptidase encoding genes,* ldt*_*Mt1*_,* ldt*_*Mt2*_, and* dacB2*, harboured three mutations, two mutations, and one mutation, respectively, but no correlation was found between these mutations and drug resistance.

**Conclusion:**

The activity of *β*-lactams against MTB and different synergetic effect of *β*-lactamase inhibitors were indicated. TBM/CLA exhibited the most activity and has a great prospect in developing novel anti-TB regimen; however, further clinical research is warranted. Moreover, the resistance to the *β*-lactam antibiotics might not be conferred by single target mutation in MTB and requires further studies.

## 1. Introduction

Tuberculosis (TB) is a great threat to the global public health that caused an estimated 1.3 million deaths in 2016 [1]. Drug resistance has been a major obstacle in the progress of eliminating TB, especially due to the emergence of multidrug-resistant (MDR) and extensively drug-resistant (XDR) MTB worldwide [2–4]. With the rising incidence of drug-resistant TB, the traditional first-line and second-line anti-TB drugs fail to fulfill the current needs of TB treatments. In such cases, developing novel drugs or drug regimens is urgent and necessary for effective control of TB. Although significant progress has been made in identification of several new anti-TB compounds, such as bedaquiline, delamanid, and pretomanid, which are in phase II or III trials [5, 6], long duration and high expenses lead to slow development of new drugs. Repurposing the currently used antibiotics may contribute to the development of novel anti-TB regimens.

*β*-lactams disrupt the cell wall biosynthesis of bacteria by preventing the formation of peptide cross-links between the adjacent polysaccharide chains in the peptidoglycan layer [7]. In* Mycobacterium tuberculosis *(MTB), two kinds of transpeptidase, the L,D-transpeptidase (mainly encoded by* ldt*_*Mt1*_ and* ldt*_*Mt2*_) and the D,D-transpeptidase (encoded by* dacB2*), catalyze the cell wall biosynthesis [8, 9]. The L,D-transpeptidase, which is responsible for the formation of over 80% transpeptide linkages (3→3 linkages) in MTB, is the target of carbapenems [10–12]. The D,D-transpeptidase that mainly catalyzes the formation of 4→3 linkages is a kind of penicillin-binding proteins (PBPs), which can be inhibited by meropenem (MEM) [13].

The resistance to *β*-lactam antibiotics encompasses the activity of *β*-lactamases. Thousands of different *β*-lactamases have been identified in various environmental and pathogenic species of bacteria [14]. MTB is intrinsically resistant to *β*-lactams due to the expression of endogenous *β*-lactamase BlaC active against a broad spectrum of *β*-lactams. CLA, AVI, and SUB are *β*-lactamase inhibitors that inhibit the hydrolysis of *β*-lactams. Recent studies have reported that a combination of the *β*-lactams and *β*-lactamase inhibitors is effective against MTB* in vitro* and* in vivo *[15–17]. The efficacy, safety, and tolerability of carbapenems, such as MEM, imipenem, and ertapenem (ETP), against MTB have been evaluated in several clinical studies [18]. Amoxicillin/clavulanate (AMX/CLA) combination has been evaluated to be efficient against MTB* in vitro* and is thus categorized into Group 5 anti-TB drugs by WHO [19, 20]. This suggests that combinations of *β*-lactams and *β*-lactamase inhibitors are prospective candidates in developing more effective treatment against TB.

In this study, to determine the most active and applicable combination of *β*-lactam and *β*-lactamase inhibitors, the* in vitro* anti-TB activity of nineteen *β*-lactams belonging to different subclasses either alone or in combination with different *β*-lactamase inhibitors was tested using drug susceptibility tests. A total of 122 MTB clinical isolates collected from China were used. Mutations in* blaC* and three main *β*-lactams targets,* ldt*_*Mt1*_,* ldt*_*Mt2*_, and* dacB2,* were analyzed to determine the relationship between drug target mutations and *β*-lactam resistance. In addition, T184A mutation in an unannotated gene,* crfA*, reported to confer carbapenem resistance in MTB was also detected.

## 2. Materials and Methods

### 2.1. Strains

A total of 122 identified clinical MTB isolates, including 47 susceptible (S), 64 MDR, and 11 XDR isolates, used in this study were collected from seven provincial TB hospitals of China in Beijing, Anhui, Fujian, Guizhou, Henan, Xinjiang, and Sichuan. All the strains were preserved in the strain bank of National Institute for Communicable Disease Control and Prevention, Chinese Center for Disease Control and Prevention.

### 2.2. *β*-Lactams and *β*-Lactamase Inhibitors

MEM was purchased from Sigma-Aldrich Co. (St Louis, MO, USA). Tebipenem, doripenem, biapenem, ertapenem, amoxicillin, ampicillin, methicillin, oxacillin, cloxacillin, nafcillin, dicloxacillin, piperacillin, cephalexin, cefuroxime, cefixime, cefpirome, aztreonam, and moxalactam and three *β*-lactamase inhibitors, CLA, AVI, and SUB, used in this study were purchased from MCE Co. (Monmouth Junction, NJ, USA) (Table 1).

### 2.3. Drug Susceptibility Test

Drug susceptibility test was performed by broth microdilution method using 96-well microplate. Briefly, a loop of MTB culture in the late log phase was fully ground and adjusted with saline to a cell density of 3 × 10^8^ cells/mL (1 McFarland standard). The suspension was diluted 1 : 20 with 7H9 broth (Difco, Detroit, MI, USA) containing 10% OADC (BD, Franklin Lakes, NJ, USA). Six groups of serial twofold drug dilutions were prepared in the 7H9 broth containing either five different constant concentrations of each *β*-lactamase inhibitor or no inhibitor at all. The final reaction mix contained 100 *μ*l of drug solution and equal volume of bacterial suspension in each well. The plates were sealed and incubated at 37°C for 7 days. Then the indicator (20 *μ*l Alamar Blue mixed with 50 *μ*l 5% Tween-80) was added to the drug-containing group when the drug-free control showed color change (blue to pink). The lowest drug concentration that inhibited the strain growth and prevented color change was recorded as the MIC value. Drug susceptibility tests for each strain were repeated twice.

The final concentrations of tebipenem ranged from 0.125 to 16 *μ*g/ml, and for the other carbapenems, 0.25 to 32 *μ*g/ml concentrations were tested in drug susceptibility tests. Ampicillin (AMP) and cefuroxime (CXM) were used at concentrations ranging from 0.5 to 64 *μ*g/ml and the other chemicals were tested at concentrations ranging from 1 to 128 *μ*g/ml. The final concentration of each *β*-lactamase inhibitor was 0.625 *μ*g/ml, 1.25 *μ*g/ml, 2.5 *μ*g/ml, 5 *μ*g/ml, and 10 *μ*g/ml, respectively.

### 2.4. Polymerase Chain Reaction (PCR) and Sequencing

Genomic DNA was isolated from mycobacterial cultures by boiling method and was used for amplification of* blaC*,* ldt*_*Mt1*_,* ldt*_*Mt2*_,* dacB2*, and* crfA* by polymerase chain reaction (PCR). The primer sets and conditions for amplification are shown in Table 2. The PCR products were sequenced (TSINGKE Biological Technology, Beijing, China) and the gene polymorphisms were identified by aligning with the reference strain H37Rv (GenBank accession no.** NC_000962**) using MEGA 7.0 software.

## 3. Results

### 3.1. Determination of Inhibitors' Concentrations

The effective concentration of each *β*-lactamase inhibitor was determined by drug susceptibility tests against six MTB clinical isolates with nineteen *β*-lactams either alone or in combination with five different concentrations of *β*-lactamase inhibitors. The results showed that the MICs did not change when 5 *μ*g/ml concentration of inhibitors was used (Table 3). Thus, we decided to use inhibitors at a fixed final concentration of 5 *μ*g/ml in the subsequent experiments. All the carbapenems, AMX, ampicillin (AMP), and cefuroxime (CXM) were selected to perform further tests, because the other *β*-lactams showed no activity against MTB in the initial screening.

### 3.2. MIC Profiles of *β*-Lactams against Clinical Isolates

The MIC profiles of the clinical isolates are shown in Table 4. In the case of carbapenems, TBM and biapenem (BIA) showed better activity with MIC_90_ of 16 *μ*g/ml when they were used alone. The MICs of carbapenems were greatly reduced (4-8 folds), except for ETP (1-4 folds), in the presence of *β*-lactamase inhibitors. TBM in combination with CLA showed the most inhibitory activity with MIC_50_ and MIC_90_ of 1 *μ*g/ml and 2 *μ*g/ml, respectively, followed by BIA (2 *μ*g/ml and 4 *μ*g/ml) and doripenem (DOR) (2 *μ*g/ml and 8 *μ*g/ml). MEM in combination with CLA also exhibited good activity with MIC_50_ and MIC_90_ of 2 *μ*g/ml and 8 *μ*g/ml, respectively, but it was lower than that of TBM and BIA. ETP in combination with any of the inhibitors showed the least activity with MIC_90_ of >16 *μ*g/ml. In the case of penicillins and cephalosporins, AMX/CLA showed highest activity against MTB with MIC_50_ and MIC_90_ of 2 *μ*g/ml and 8 *μ*g/ml, respectively, followed by AMP/CLA (4 *μ*g/ml and 16 *μ*g/ml). CXM alone or in combination with any of the inhibitors showed no anti-TB activity even when the highest drug concentration was tested.

Comparison of the synergistic effect of the three different *β*-lactamase inhibitors shows that CLA exhibited the most potent activity in inhibiting the *β*-lactamase. Overall, the MICs of *β*-lactams were reduced most prominently (4-32 folds) in the presence of CLA, followed by AVI (4-8 folds). However, the least activity was observed in case of SUB as MIC_50_ and MIC_90_ of all the tested drug combinations with SUB showed lesser reduction (2-8 folds) than that in the presence of CLA and AVI, especially when used in combination with TBM, BIA, and AMP.

### 3.3. Relationship between Drug Target Mutation and Resistance

Since the *β*-lactamase BlaC, L,D-transpeptidase, and D,D-transpeptidase are potential targets of *β*-lactams, the encoding genes, including* blaC*,* ldt*_*Mt1*_,* ldt*_*Mt2*_, and* dacB2*, were analyzed to illustrate the role of drug target mutations in the emergence of the *β*-lactams resistance in MTB. In addition, mutation at position T184A in an unannotated protein encoding gene,* crfA*, which was earlier reported to confer carbapenem resistance in MTB [21], was also analyzed. Mutations in the five genes are shown in Table 5 and the MIC profiles of the mutant strains are listed in the supplementary materials. As observed, 16.4% (20/122) of clinical isolates carried 8 mutation sites in* blaC*. The silent mutation, C786T (11 isolates), was the most predominant mutation. Three nonsynonymous mutations, T333G, T492A, and G514A, were observed in* blaC*, while none of these mutations was related to *β*-lactam resistance. Interestingly, one strain, GZ10116, which carried G514A mutation, was highly susceptible to *β*-lactams irrespective of the presence of inhibitors.

There were 1, 1, and 2 strains harbouring A111G, G448A, and C659T mutations, respectively, on* ldt*_*Mt1*_. 1 strain and 4 strains, respectively, harboured C594T and A776G mutation in* ldt*_*Mt2*_. Only one strain, which harboured A776G mutation in* ldt*_*Mt2*_, was resistant to all the combinations of *β*-lactams and inhibitors. However, we did not find any mutation associated with the *β*-lactam resistance in *ldt*_*Mt*1_  and* ldt*_*Mt2*_. Only one mutation, T659A, was found on* dacB2 *in four strains, but it was also not associated with the *β*-lactam resistance. In addition, the results showed that no mutation was detected in c*rfA *among all the strains in our study. We also did not find any strain harbouring mutations in the* blaC* and transpeptidase genes simultaneously.

## 4. Discussion

Novel drugs or regimens are urgently needed due to the rise in the prevalence of multidrug-resistant TB worldwide. *β*-lactams are widely used and well-tolerated antibiotics used for controlling clinical Gram-positive and Gram-negative bacterial infections [22]. Recently, the combination of *β*-lactamase inhibitor and *β*-lactam was shown to be effective in restoring the potency of *β*-lactam against MTB [15–17]. In the present study, nineteen commercially available *β*-lactams and three *β*-lactamase inhibitors were screened* in vitro *to identify the most potent combination against MTB. The results show that carbapenems, except for ETP, exhibited excellent activity against MTB clinical strains. This may be due to the inactivation of multiple enzymes by carbapenems, whereas the other *β*-lactams did not inhibit the L,D-transpeptidase in MTB [10]. The activity of AMX was also improved notably in the presence of clavulanate. While the efficacy of AMX/CLA against clinical TB remains controversial, carbapenems may serve as an optimal alternative in developing novel anti-TB regimens. The results showed that all the tested cephalosporins in our study were inactive against MTB. Dincer* et al*. [23] reported that cefazolin in combination with CLA was active against H37Ra. This may be due to the different strain tested and different methodology used. Additionally, the concentration of CLA, that is, 32 *μ*g/ml, was much higher than the concentration (5 *μ*g/ml) used in our test. This suggests that a more universal drug susceptibility testing protocol for determining the MIC of *β*-lactams against MTB is needed in the future.

Carbapenems displayed a variable activity in this study. TBM in combination with CLA exhibited the highest activity against MTB, followed by BIA and DOR. MIC_90_ of TBM/CLA was 2 *μ*g/ml, which can be easily obtained in plasma with the administration of a single dose of 200 mg TBM pivoxil granules [24]. Comparable activity to that of TBM/CLA was observed in case of BIA/CLA. The maximum plasma concentration (*C*_max_) of BIA after a single intravenous dose was 17.4 *μ*g/ml [25], which exceeded MIC_90_ (2 *μ*g/ml) of BIA/CLA. Both of BIA and TBM are stable against renal dehydropeptidase-I and are also safe for central nervous system, since they do not exhibit competitive binding to the *γ*-aminobutyric acid (GABA) receptors [26]. However, BIA requires intravenous administration that is not easily complied with by the patients. MEM/CLA was previously reported to be effective against MDR-TB. The *C*_max_ and AUC values of MEM were 26 *μ*g/ml and 27.2–32.4 *μ*g·h/ml, respectively, after being administered by an intravenous infusion of 500 mg of MEM [27]. In our results also, MEM/CLA exhibited potent activity (MIC_90_, 8 *μ*g/ml) against MTB but it was weaker than that of TBM/CLA, which is consistent with previous study [28]. This may be explained by lower *K*_m_ and less breakdown of the covalent tebipenem-BlaC adduct, in contrast to that of MEM, thus suggesting a higher activity of TBM against MTB [17, 29]. Oral administration of the TBM pivoxil had a better protective effect than meropenem in all the tried sepsis models, due to its greater tissue distribution [30]. Hence, as an oral carbapenem, TBM may be a better option in developing new anti-TB regimen.

MTB harbours a chromosomally encoded *β*-lactamase, which is selectively susceptible to *β*-lactamase inhibitors. In this study, varied synergistic effects of three different *β*-lactamase inhibitors were observed. Consistent with previous study [28], the greatest reduction in MIC was achieved by CLA in combination with *β*-lactams, especially in AMX/CLA (32-fold in MIC_50_). AVI, a newly developed *β*-lactamase inhibitor, combining with ceftazidime exhibited good activity against* Enterobacteriaceae* and* Pseudomonas aeruginosa *[31]. However, a combination of AVI with *β*-lactams displayed a lesser reduction in MIC of *β*-lactams compared to CLA. A recent report has demonstrated that the efficiency of AVI was lower than that of CLA due to its bulky rings that may influence its binding to BlaC [32]. The unstable covalent adduct of SUB and BlaC may also explain its lesser inhibiting activity than that of CLA [33]. Thus, CLA seems to be the most suitable in developing anti-TB regimens encompassing *β*-lactams. In addition, the concentration of 5 *μ*g/ml of CLA used in the current study was lower than the *C*_max_ of 5.9 *μ*g/ml achieved after a 250 mg three times a day dosage, suggesting that it can be easily achieved in clinical use [34].

For investigating the relationship between mutations in the *β*-lactam targets and the drug susceptibility of MTB clinical isolates,* blaC*,* ldt*_*Mt1*_,* ldt*_*Mt2*_, and* dacB2* in 122 MTB clinical isolates were sequenced and analyzed. BlaC hydrolyzes the *β*-lactam ring, which is essential for the activity of *β*-lactam antibiotics, resulting in the *β*-lactams resistance in MTB. Eight different mutations were observed in* blaC*. However, we did not find any association between these mutations and *β*-lactam resistance. Interestingly, one of the strains harbouring G514A (Gly172Ser) mutation was highly susceptible to *β*-lactam antibiotics in either the presence or the absence of the inhibitors. Zhang* et al*. [35] have reported that the S111R mutation on BlaC may be associated with an increased susceptibility of MTB. Considering that the mutation Gly172Ser may affect the interaction of BlaC and *β*-lactams [36], we may speculate that this amino change leads to a conformation change in BlaC, which influences its activity.

Mutation on target gene often confers drug resistance to bacteria. Four clinical MTB strains were found to carry mutation A776G in* ldt*_*Mt2*_ and two of them were susceptible to *β*-lactams. Similarly, among the* dacB2* mutants, one exhibited resistance, while the other exhibited susceptibility to *β*-lactams. However, we did not find any correlation between these mutations and the *β*-lactams resistance. In this study, we only tested four potential targets and none of the strains harboured mutations simultaneously on all the four genes. It is reported that the *β*-lactam antibiotics have to bind to at least three of the four identified PBPs to be effective [37]. Considering the multiple targets of *β*-lactams, we may speculate that the drug resistance may not be conferred by mutation in a single target. Further studies are required to demonstrate the role of drug target mutations in the development of *β*-lactam resistance in MTB in order to consider the inclusion of *β*-lactams in the anti-TB treatment regimens.

## 5. Conclusions

In summary, varied activities of carbapenems, penicillins, and cephalosporins alone or in combination with *β*-lactamase inhibitors against MTB were observed. TBM in combination with CLA showed the most remarkable activity and has a good prospect in developing novel anti-TB regimens. Further studies are warranted to investigate the efficacy of TBM/CLA in the clinical trials. Additionally, resistance to the *β*-lactams may not be conferred by single target mutation in MTB and thus requires further investigation.

## Figures and Tables

**Table 1:** The *β*-lactams and *β*-lactamase inhibitors for drug susceptibility tests.

Classification	Agent	Generation
Carbapenem	Tebipenem	
	Meropenem	
	Doripenem	
	Biapenem	
	Ertapenem	
Penicillin	Amoxicillin	
	Ampicillin	
	Methicillin	
	Oxacillin	
	Cloxacillin	
	Nafcillin	
	Dicloxacillin	
	Piperacillin	
Cephalosporin	Cephalexin	First generation
	Cefuroxime	Second generation
	Cefixime	Third generation
	Cefpirome	Fourth generation
Monobactam	Aztreonam	
Oxacephem	Moxalactam	
*β*-lactamase inhibitor	Clavulanate	
	Avibactam	
	Sulbactam	

**Table 2:** Primers and conditions used for amplification and sequencing.

Primers	Sequence (5′-3′)	PCR conditions			Product length (bp)
		Denaturation^a^ (s)	Annealing (°C, s)	Elongation^b^ (s)	
BlaC-F	ATGCGCAACAGAGGATTCGGTC	30	61, 30	65	924
BlaC-R	CTATGCAAGCACACCGGCAACG				
Ldt_Mt1_-F	ATGCGTCGAGTGGTTCGTTATC	30	58, 30	55	756
Ldt_Mt1_-R	CTAGCCGACCACCTCAATGG				
Ldt_Mt2_-F	ATGCCAAAGGTGGGGATTGC	30	59, 30	90	1227
Ldt_Mt2_-R	TTACGCCTTGGCGTTACCGGC				
DacB2-F	ACCAGCAACTGCTGGATTTC	30	60, 30	85	1196
DacB2-R	CGTTGATGACCAACGTCTTC				
CrfA-F	ACCCGGCTCACAGAGAATCG	30	60, 30	40	457
CrfA-R	TATCACCGGTAGGCCATGC				

Note: ^a^: denaturation temperature was 94°C; ^b^: elongation temperature was 72°C. All PCRs were performed for 31 cycles and each reaction was initialized by denaturation at 94°C for 10 min and terminated with a final elongation step at 72°C for 10 min.

**Table 3:** MIC profiles of *β*-lactams alone or in combination with inhibitors against six MTB isolates.

Inhibitors	Concentration	MIC range (*μ*g/ml)																		
		TBM	MEM	DOR	BIA	ETP	AMX	AMP	MET	OXA	CLO	NAF	DIC	PIP	LEX	CXM	CFM	CPO	ATM	MOX
Clavulanate	0	8-16	16-32	8-16	8-16	32->32	64->128	>64	>128	128->128	>128	>128	>128	>128	64->128	>64	>128	64->128	>128	>128
	0.625	<1-2	4-8	8-16	8-16	16-32	16-32	32-64	128->128	128->128	128->128	>128	>128	>128	64->128	32->64	>128	64->128	>128	>128
	1.25	<1	4	4-8	4-8	16-32	8-32	16-64	64->128	128->128	128->128	>128	>128	>128	32->128	16-64	>128	32->128	>128	>128
	2.5	<1	2-4	4-8	2-4	8-32	4-32	4-64	32->128	64->128	128->128	>128	>128	>128	32->128	16-32	>128	32->128	>128	>128
	5	<1	2-4	2-4	1-2	8-16	4-8	<1-32	16->128	32->128	64->128	64->128	>128	64->128	16->128	4-16	>128	16->128	>128	>128
	10	<1	2-4	2-4	1-2	8-16	4-8	<1-32	16->128	32->128	64->128	64->128	>128	64->128	16->128	4-16	>128	16->128	>128	>128
Avibactam	0	8-16	16-32	8-16	8-16	32->32	64->128	>64	>128	128->128	>128	>128	>128	>128	64->128	>64	>128	64->128	>128	>128
	0.625	4-8	8-16	8-16	8-16	16->32	64-128	32-64	128->128	128->128	128->128	>128	>128	>128	32->128	32->64	>128	32->128	>128	>128
	1.25	2-4	8-16	4-16	8-16	16-32	32-128	16-64	128->128	128->128	128->128	>128	>128	>128	32->128	32-64	>128	32->128	>128	>128
	2.5	<1-4	8-16	4-8	4-8	8-32	32-64	8-64	64->128	64->128	128->128	>128	>128	>128	32->128	16-32	>128	32->128	>128	>128
	5	<1-2	4-16	2-4	2-4	8-16	8-32	8-32	32->128	64->128	128->128	64->128	>128	>128	32->128	8-32	>128	32->128	>128	>128
	10	<1-2	4-16	2-4	2-4	8-16	8-32	4-32	32->128	64->128	128->128	64->128	>128	>128	32->128	8-32	>128	32->128	>128	>128
Sulbactam	0	8-16	16-32	8-16	8-16	32->32	64->128	>64	>128	128->128	>128	>128	>128	>128	64->128	>64	>128	64->128	>128	>128
	0.625	8-16	16-32	8-16	8-16	32->32	64->128	32-64	128->128	128->128	>128	>128	>128	>128	32->128	32->64	>128	32->128	>128	>128
	1.25	4-16	16-32	8-16	4-16	16-32	64->128	16-64	64->128	64->128	128->128	>128	>128	>128	32->128	32-64	>128	32->128	>128	>128
	2.5	4-8	16-32	4-16	4-8	16-32	32->128	8-64	32->128	64->128	128->128	64->128	>128	>128	32->128	16-32	>128	32->128	>128	>128
	5	2-8	8-32	4-8	2-8	8-32	16->64	4-64	16->128	32->128	128->128	64->128	>128	>128	32->128	8-32	>128	32->128	>128	>128
	10	2-8	8-32	4-8	2-8	8-32	16->64	4-64	16->128	32->128	64->128	64->128	>128	>128	32->128	8-32	>128	32->128	>128	>128

Note: TBM, tebipenem; MEM, meropenem; DOR, doripenem; BIA, biapenem; ETP, ertapenem; AMX, amoxicillin; AMP, ampicillin; MET, methicillin; OXA, oxacillin; CLO, cloxacillin; NAF, nafcillin; DIC, dicloxacillin; PIP, piperacillin; LEX, cephalexin; CXM, cefuroxime; CFM, cefixime; CPO, cefpirome; ATM, aztreonam; MOX, moxalactam.

**Table 4:** MICs of *β*-lactams alone or in combination with a fixed concentration of 5 *μ*g/ml of the inhibitor against 122 MTB isolates.

Antibiotic	Inhibitor	MIC range (*μ*g/ml)	MIC_50_ (*μ*g/ml)	MIC_90_ (*μ*g/ml)
TBM	None	0.5->16	8	16
	CLA	0.125->16	1	2
	AVI	0.125->16	1	2
	SUB	0.5->16	2	4
BIA	None	2->32	8	16
	CLA	0.25->32	2	4
	AVI	0.5->32	2	4
	SUB	0.5->32	4	8
DOR	None	2->32	16	16
	CLA	0.5->32	2	8
	AVI	0.5->32	4	8
	SUB	0.5->32	4	16
MEM	None	4->32	>32	>32
	CLA	0.25->32	2	8
	AVI	1->32	4	16
	SUB	4->32	8	16
ETP	None	8->32	>32	>32
	CLA	2->32	8	16
	AVI	4->32	8	16
	SUB	4->32	16	32
AMX	None	8->64	>64	>64
	CLA	<0.5-32	2	8
	AVI	<0.5-64	8	16
	SUB	2->64	8	32
AMP	None	32->64	64	>64
	CLA	0.5->64	4	16
	AVI	0.5->64	8	16
	SUB	0.5->64	16	32
CXM	None	16->64	>64	>64
	CLA	1->64	32	>64
	AVI	2->64	32	>64
	SUB	4->64	64	>64

Note: MIC_50/90_, MICs that inhibit 50% and 90% of the isolates, respectively.

**Table 5:** Mutations on *blaC*, *ldt*_*Mt1*_, *ldt*_*Mt2*_, and *dacB2* genes.

Gene	Substitution	Amino acid change	Number of mutants
*blaC*	G183A	Leu61Leu	1
	T333G	Ser111Arg	3
	C354T	Ser118Ser	1
	G486C	Ala162Ala	1
	T492A	Phe164Leu	1
	G514A	Gly172Ser	1
	C610T	Leu204Leu	1
	C786T	Ile262Ile	11
*ldt*_*Mt1*_	A111G	Pro37Pro	1
	G448A	Ala150Thr	1
	C659T	Ala220Val	2
*ldt*_*Mt2*_	C594T	Gly198Gly	1
	A776G	Asp259Gly	4
*dacB2*	T659A	Leu220Gln	4

## Data Availability

The datasets used to support the findings of this study are available upon request from the corresponding author.

## Supplementary Materials

Supplementary Materials The details of mutations and the MIC profile of each mutant in this study are listed in the supplementary material file.
